# Comparison of the Bending Behavior of Cylindrically Shaped Lattice Specimens with Radially and Orthogonally Arranged Cells Made of ABS

**DOI:** 10.3390/polym16070979

**Published:** 2024-04-03

**Authors:** Katarina Monkova, Peter Pavol Monka, Adrián Vodilka

**Affiliations:** 1 Faculty of Manufacturing Technologies with a Seat in Prešov, Technical University in Kosice, 080 01 Presov, Slovakia; peter.pavol.monka@tuke.sk (P.P.M.); adrian.vodilka@tuke.sk (A.V.); 2 Faculty of Technology, Tomas Bata University in Zlin, Nam. T.G. Masaryka 275, 760 01 Zlin, Czech Republic

**Keywords:** lattice structure, cell arrangement, bending behavior, ABS plastics, ductility index

## Abstract

The article deals with the comparison of the bending behavior of cylindrical lattice samples with radially and orthogonally arranged cells made of ABS material. The structures were designed in PTC Creo Parametric 8 software, while four types of lattice structures were evaluated: Rhombus, Cuboidal BCC, Octagon, and Starry, in three material volume fractions: 44, 57, and 70%, together with tubular and rod-shaped samples. The Fused Filament Fabrication (FFF) technique was chosen for the production of ABS plastic samples. Based on the bending tests, the dependences of the force on the deflection were recorded and the obtained data were statistically processed to identify outliers using the Grubbs test. The maximum stresses were calculated and the dependences of the stresses on the volume fractions were plotted. Along with energy absorption, ductility indices were also specified. Although the Rhombus structure appears to be the best based on the ductility indices obtained, on the other hand, the structure showed the lowest values of bending stresses (in the range from 10.6 to 12.6 MPa for volume fractions ranging from 44 to 70%, respectively). Therefore, from a synergic point of view of both factors, stress and ductility, the Starry structure exhibits the best flexural properties among those investigated.

## 1. Introduction

Porous solids are all around us. They can be natural (like plants, meat, rocks, stones, soils, etc.), as well as man-made (like gravels, cement, concrete, plaster, filters, gels, and others). The human body itself, which is an assembly of bones, muscles, skin, and so on, is also a complex structure made of porous solids. Since porous solids are everywhere, they affect our day-to-day life. The concept of porous materials has been known for a number of years, but its behavior is far less understood than that of other materials. By the end of the 20th century, studies on porous materials had made a number of important discoveries thanks to additive technologies that enabled researchers to produce, on a macroscale, porous material of complex shapes, and thus investigate both their functional and structural characteristics. For functional applications, the physical properties must be known, whereas for structural applications, the basic mechanical properties are also considered, and vice versa. For the simultaneous requirements of both types of applications, the physical and mechanical properties are of equal importance [1,2,3].

The manufacturability of cellular materials has improved thanks to the fast development of additive technologies, which has brought many advantages into technical practice on one hand, but on the other hand there are still many limitations and disadvantages that need to be gradually eliminated. Restrictions are most often connected with the type of material and associated technologies. For metallic materials, Direct Metals Laser Sintering (DMLS) technology is currently the most commonly used, and for plastic materials, it is the Fused Filament Fabrication (FFF) technique.

With DMLS technology, it is possible to achieve the production of components with much higher quality and precision, which brings great advantages in the production of finer porous structures. Due to the laser beam used, the height of the layer is much smaller than with FFF technologies, and therefore, the staircase effect is not so visible. There is no heterogeneity of properties as there is with FFF technology, where the mechanical properties of the manufactured components in the direction of application of the layers are much lower due to the low adhesion forces between the layers. On the other hand, DMLS technology is much more expensive, both in terms of the prices of powder materials and in terms of the procurement and operating costs of the production equipment. While structures with closed pores can also be produced with FFF technology (if they are self-supporting structures without the need to create supports), this is not possible with DMLS technology, as the powder material would remain closed in the pores and it would not be possible to remove it.

There are various types of porous material that can give a component many advantages, demonstrated by comprehensive physical and mechanical properties, which primarily depend on a basic material followed by pore topology, pore sizes, or their distribution [4,5]. Such lightened materials can be used in the aerospace, electronics and communication, transportation, atomic energy, medical, environmental protection, metallurgy, machine, construction, electrochemistry, petrochemical, and bioengineering industries. This is due to their desirable capabilities, such as flow separation/filtration, distribution, sound absorption and noise reduction, dampening, electromagnetic screening, heat insulation and fire resistance, heat exchange, catalysis, electrochemical process, and medical plastic and repair [6,7,8,9].

The properties of porous materials with deterministically distributed voids can be better predicted, while they can be divided into two categories. The first one includes the lattice structures with a strut architecture and the second one is based on a complex surface topology, mostly with so the so-called Triply Periodic Minimal Surfaces (TPMS) architecture [10,11,12,13].

Except for a lattice type structure, another very important characteristic of porous materials is so-called porosity *P* or volume fraction *Vf*. If the total volume of the sample is denoted as *V*, the volume of the solid phase is defined as *Vs*, and the volume of the pore phase (holes) is *Vp*, while *V = Vs* + *Vp*, then the volume fraction of the porous phase is commonly called porosity and denoted by *P* = *Vp*/*V*. However, volume fraction of a solid phase *Vf* is a normalized variable that is generally more useful, and it is then given as (1 *− P*). It can be also written as

*Vf* = *Vs*/*V*. (1)

The behavior of porous cellular lattice structures has recently become the subject of many studies, but only a few of them have focused on investigating the properties of lattice structures under bending.

Huan Jiang and his team designed a new type of tubular lattice architecture by rolling planar lattice structures with a negative Poisson’s ratio, and subsequently fabricated samples using the FDM method. The large-strain bending behavior was investigated using a combined experimental and numerical approach. The goal of the research was to design a new type of lattice structure with improved properties. The authors investigated the bending behavior of auxetic tubular lattice (ATL) and conventional diamond tube lattice (DTL) structures using a combined experimental and numerical approach. They found that ATL exhibits a more yielding behavior and the ductility increased up to 85.4% compared to the DTL structure. During the research, it was found that the advantage of ATL over DTL is higher ductility, higher yield, more local bending, and excellent global stability. Thanks to these properties, ATL can be applied in several areas, such as medicine (bone replacements, bio-implants) and robotics (robotic arms, drone production) [14].

Sandwich structures with aluminum face plates and 3D-printed lattice cores with 2D and 3D topology were the topics of Zhaobing Liu’s research. Numerical simulations and experimental measurements were performed to evaluate the mechanical properties of these constructions. The results of the study showed that sandwich structures with 3D face-centered and cubic-centered cells exhibit better mechanical properties than sandwich structures with 2D cells. In conclusion, the authors point out that sandwich structures with six different 3D-printed lattice core topologies show a significant improvement in mechanical properties compared to traditional sandwich structures, namely better resistance to damage (especially in the area of elastic deformation), higher load capacity, and better energy absorption [15].

The goal of Gullapalli’s research was to design and test rectangular struts of cellular lattice structures with different unit cell configurations to determine the structural design for the best flexural performance in engineering applications. The results of the study have shown that cellular lattice structures based on triangular and honeycomb shapes exhibit maximum bending strength. The triangular structure was also shown to have the highest flexural modulus value among all five cellular lattice structures. The study also found that higher porosity of honeycomb structures results in higher flexural strength and flexural modulus and shorter construction time up to 61% porosity [16].

Dorin Catana’s study pointed out that for additively manufactured samples from the studied filaments or their combination, the results of the simulation process are close to the results of experimental bending tests. For the bending stress in the case of rod-type specimens, the deviations of the simulation results compared to the test results were between 7.2 and 5.7%. In the case of bending deflections (displacements), these deviations were between 17.3 and 8.5%. In the case of 3D-printed samples obtained by connecting two filaments, the average deviation was around 5% (depending on the simulation method) for bending strength and around 10% for displacements. Based on these results, it can be concluded that the simulation process can be applied with good results on 3D-printed PLA structures under bending stress [17].

Meltem Eryildiz dealt with the design of new core topologies of sandwich structures to improve their bending properties. Six independently designed core topologies were produced from polylactic acid (PLA) by the fused deposition modeling (FDM) additive manufacturing method and they were numerically analyzed, while the structure with the triple bow core exhibited the highest mechanical properties (stiffness and strength) [18].

The influence of various geometrical parameters on the flexural properties of 3D-printed honeycomb structures and their sandwich panels with face layers made of E-glass/epoxy laminates was investigated by Pirouzfar and Zeinedini. This research found out that the flexural properties of 3D-printed honeycomb samples and their laminated composite sandwich panels can be significantly improved by changing the cell wall thickness, honeycomb core cell direction, and core material. The largest values of the normalized stresses in the bending of the face layer and the shear stresses of the core corresponded to the horizontal core sandwich panel with a cell wall thickness of 2 mm [19].

In the study of Öteyaka, the effects of different patterns and infill rates on the vibration-damping ability of 3D-printed materials were investigated. The researchers produced 3D-printed materials with four different patterns and five different filling rates. The research showed that increasing the filler ratio leads to an increase in flexural strength and flexural modulus, but only to a certain extent. The best results were achieved at a fill ratio of 20%, with the fill pattern having a significant effect on vibration-damping properties. The cross pattern showed the best result, followed by the tri-hexagon pattern and the grid pattern. So, for most applications, the optimum filler ratio is 20%, while a suitable selection of the filler pattern can improve the vibration-damping properties of additively manufactured plastic parts to a large extent [20].

A 2022 study by Fongsamootr and his team investigated the out-of-plane bending behavior of two-dimensional (2D) periodic plates. The researchers used numerical simulations and experiments to compare the bending behavior of plates with different unit cell topologies. The authors proposed a new method to improve the mass efficiency of 2D periodic plates. One of the important findings of this research was that the flexural moduli obtained from FEM models of periodic structures are in good agreement with values calculated from FEM models of equivalent homogeneous plates composed of materials with effective out-of-plane elastic properties [21].

In recent years, porous materials have been developed rapidly in terms of the preparation and characterization of the physical and mechanical properties, mainly thanks to additive technology. There are many applications for porous materials due to their excellent overall performance. The ultimate goal for the development of these materials and the characterization of their properties is to use them to meet specific design requirements. Therefore, characterization and testing of the materials must be conducted before any attempt to use them [22,23].

Several other studies on the properties of lattice structures could be found in already published research, but to the best of the authors’ knowledge, the selected topologies of the lattice structures (Octagon, Star, Rhombus, and Cube) designed for the presented research on the mechanical bending properties of ABS material have not been comparatively studied yet, which can be considered as a novelty. Another novel specific feature of the presented research is the cylindrical shape of the samples, which is often found in technical applications, but this shape is rarely tested in bending, as the samples are usually quadratic of the cross-section and sandwich types. The research aimed to compare the bending properties of four types of lattice structures, while the basic cells in two of them, Rhombus and Cube (specifically Body-Centered Cubic—BCC), were arranged orthogonally, and in two of them, Star and Octagon, they were arranged radially. A total of five volume fractions *Vf* of the samples made from the plastics ABSplus—P430 Ivory (Prusa Research, a. s., Prague, Czech Republic) were used in the investigation. Additionally, the behaviors of the samples with a tube shape (without structures), representing the volume fraction *Vf* = 25%, and a bar shape, representing *Vf* = 100%, were experimentally analyzed.

## 2. Materials and Methods

### 2.1. Specification of Samples

**Figure 1:**
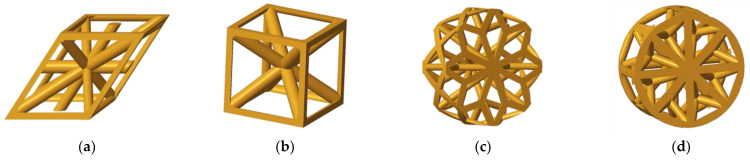
The basic cells of the structures; (**a**) Rhombus; (**b**) Cuboidal BCC; (**c**) Starry; (**d**) Octagon.

Within the presented research, the authors decided to compare the bending behavior of four types of lattice structures arranged in the core of a cylindrical shell; two of them were distributed orthogonally (Rhombus and Cuboidal BCC) and two of them radially (Starry and Octagon). The basic cells of the structures are presented in Figure 1.

**Figure 2:**
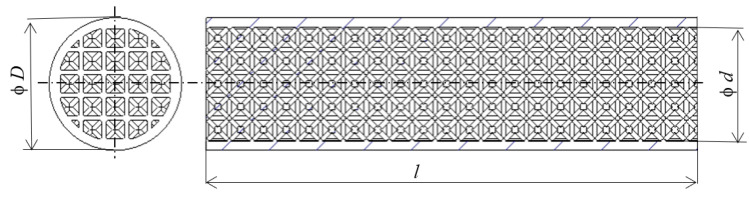
The basic sizes of a sample.

Virtual 3D models were generated in the software PTC Creo Parametric 8. The geometry of the sample was cylindrically designed so that the lightened core diameter of *d* = 25 mm was formed by a lattice structure, which was covered with a 2 mm layer of continuous material on the surface. The resulting diameter *D* of the sample was therefore *D* = 29 mm, as shown in Figure 2. The length of the samples *l* = 200 mm was limited by the working space of the 3D printer Prusa i3 Mk2 (Prusa Research a.s., Prague, Czech Republic), which was chosen for this study due to its availability at the authors’ workplace.

Preliminary research showed that the minimum strut diameter produceable by the printer of the selected ABS material is 1 mm that was also the reason for setting the minimum volume fraction *Vf* = 44%, while the volume of the solid phase *Vs* for the computation of the volume fraction includes both the volume of the structure itself as well as the shell surface layer. In order to compare the results, the total volume fractions *Vf* of 44, 57, and 70% were set for each type of structure. Additionally, the behaviors of the samples with a tube shape (without structures), representing the volume fraction *Vf* = 25%, and a bar shape, representing *Vf* = 100%, were experimentally analyzed. Due to the possibility of repeating the experiments and statistical evaluation of the results, six pieces of samples of each type of structure and volume fraction *Vf* were produced (one of them was always used for preliminary tests), so in total, eighty-four pieces of samples were tested (4 types of structures × 3 volume fractions × 6 pieces + tube-type sample × 6 pieces + bar-type sample × 6 pieces). The most important characteristics of the individual basic cells are listed in Table 1, while the *x* axis is the axis of rotation of the cylindrically shaped sample and *φ* is a skewing angle.

### 2.2. Material and Fabrication

The samples were made of ABS (Acrylonitrile–Butadiene–Styrene), which is one of the most widely used and universal printing materials, with a wide range of uses, from household items and toys to the automotive industry. It is a durable amorphous thermoplastic copolymer that excels in its good resistance to mechanical damage. It is stiff, tough, resistant to low and high temperatures depending on the type, not very absorbent, and harmless to health. It is suitable for both indoor and outdoor use. ABS is a thermoplastic polymer; that means it can be melted and crystallized multiple times without degrading too much [24,25].

It is resistant to acids, alkalis, hydrocarbons, oils, and fats. It can be processed up to a temperature of 280 °C. Shrinkage then varies between 0.3 and 0.7%. The material can also be processed very well, e.g., by grinding or smoothing with acetone vapors. Glueing with solvent-based adhesives based on toluene and methylene chloride as well as polyacrylate adhesives is also possible. The softening temperature for ABS is around 100 °C [26].

White EasyABS filament (producer Prusa Research a.s., Prague, Czech Republic) with a diameter of 1.75 mm was used for the production of samples in this study. The basic characteristics given by the producer in the material sheet are listed in Table 2 [27].

The samples for the presented study were 3D-printed by the FFF technique using a Prusa i3 Mk2 3D printer (Prusa Research, a. s., Prague, Czech Republic), while during production, the samples were oriented so that the rotation axis of the cylindrical samples was perpendicular to the printed platform.

During the production of samples, however, it became clear that to ensure sufficient quality of the samples, it was necessary to find the right combination of technological parameters together with other boundary conditions (such as the strategy of creating the geometry—the movement of the print head, the position of the cell concerning the beginning of the coordinate system, and thus also the building platform, etc.). The preliminary produced samples showed many defects; moreover, in some cases, it was not even possible to complete the production of the sample.

**Figure 3:**
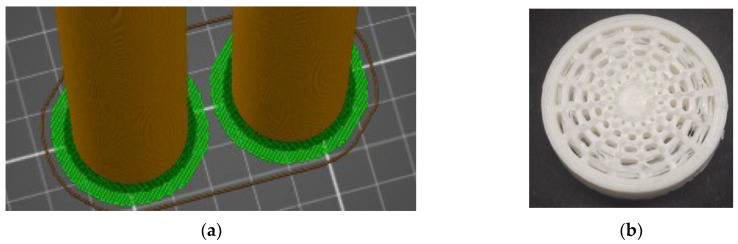
(**a**) The position of the samples during printing with a raft in the 3D printing platform; (**b**) an example of one set of produced samples with structures in the cores.

ABS is processed at a temperature of 210–250 °C, and the resulting product is stable at temperatures up to 100 °C [28]. Its disadvantage is that it shrinks and changes shape during cooling, which is very noticeable, especially with larger models. This can be partly prevented by slow cooling. Because of that, the model must remain on the heated printer mat, glued as long as possible. Another problem that follows from the previous one is the adhesion of the first layer to the substrate. This can be ensured by heating the pad to at least 90 °C [29]. To stabilize the samples, when creating the G-code in PrusaSlicer 2.5 software, a so-called raft (Figure 3a in green) was generated on the interface between the sample’s body and the printing platform. The raft is an intermediate layer between the printed object and the substrate, which ensures better adhesion of the printed object to the substrate and is significantly wider than the contact surface of the product with a pad. Two raft layers were used for printing, which ensured sufficient adhesion of the specimens even during the printing of one set of samples lasting more than 25 h [30,31,32].

The next problem that was revealed during the printing of the first samples was significant stringing (Figure 3b). It was eliminated by selecting the appropriate print strategy, print speed, and setting the correct nozzle temperature. In this research, the nozzle temperature was set to a temperature of 240 °C and the pad temperature was set to 100 °C during the production process, while the printing speed was 30 mm/s. A nozzle diameter of 0.4 mm and a layer thickness of 0.3 mm were used for the sample printing [33].

**Figure 4:**
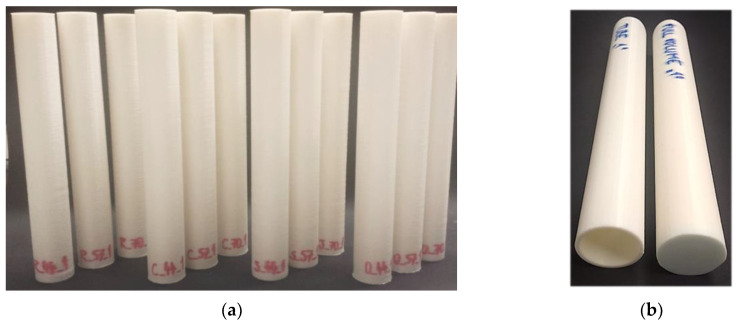
One set of printed specimens: (**a**) with a lattice structure in the core; (**b**) tube- and bar-type.

To identify the samples, the letter of the structure type (R—Rhombus; C—Cuboid BCC; S—Starry; O—Octagon) was used together with the number 44, 57, or 70, corresponding to the volume fraction and the number from the series 1–5. So, e.g., the designation S_44_2 means that it is a Starry-type sample with *Vf* = 44% from the second series. An example of a set of produced specimens is shown in Figure 4.

### 2.3. Experimental Conditions and Methodology of Evaluation

**Figure 5:**
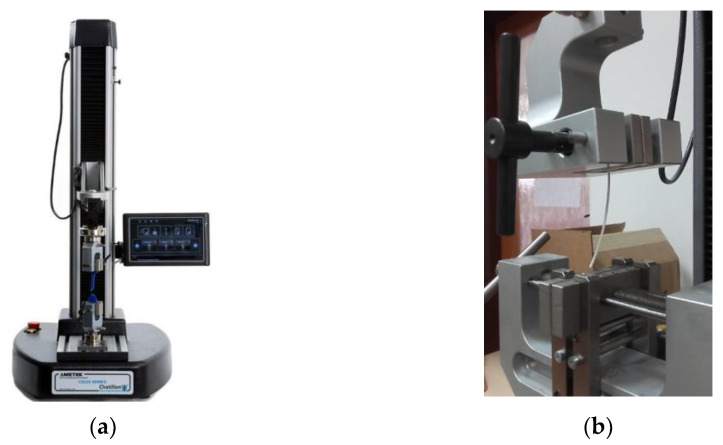
The preliminary tests of White EasyABS filament properties: (**a**) desktop tensile testing machine CS 225; (**b**) testing set.

Preliminary tests to check the filament properties were carried out according to ISO 527-1/-2 [34] standards at an ambient temperature of 20 °C and a relative humidity of 55%, while a desktop tensile testing machine CS 225 (Figure 5a) (AMETEK Lloyd Instruments Ltd., West Sussex, UK) was employed. Five repetitions of the measurements were performed (Figure 5b), and the results confirmed the values of tensile stress declared by the producer [35], and the filament for the production of samples in the research was even slightly stronger, i.e., the average value showed ultimate tensile stress of 45 MPa, while it is listed as 43 MPa in the material datasheet.

The experimental investigation of the flexural properties of the porous structures was performed according to ISO 178:2019 standard [36] via 3-point bending tests using the ZWICK 1456 machine (ZwickRoell GmbH & Co. KG, Ulm, Germany) with the software TestXpert II at an ambient temperature of 22 °C and a relative humidity of 60%. The next settings were:

- push thorn radius—5 mm;
- cross-head speed—20 mm/min;
- distance of supports—170 mm.

**Figure 6:**
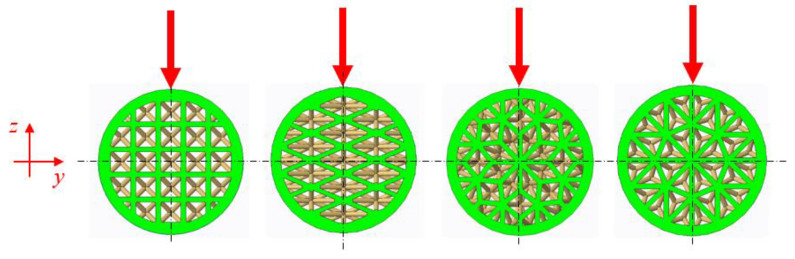
The cross-section areas of the tested samples with their orientation during the experiments with respect to the force vector induced by the push thorn of the testing machine.

Since each sample, characterized by the type of structure and the volume fraction *Vf*, was produced in a group of 6 pieces, it was necessary to ensure the same rotation of the given structure in relation to the push thorn of the testing machine in all repetitions of the experiments. For better evaluation of the results, the orientation of the structures was used so that the axis of the push thorn was in line with the “*z*” axis of the CAD model of the sample prepared in the software PTC Creo Parametric 8, as shown in Figure 6.

**Figure 7:**
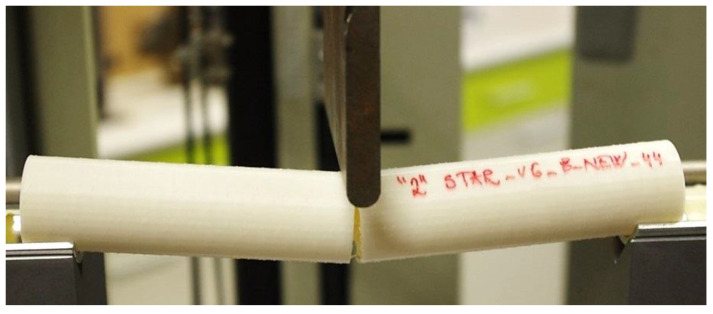
Bending test performance.

Special semi-cylindrical jigs were made to secure the samples against rotation and slippage, while the distance between the supports was 170 mm. The experimental set up for the bending test is shown in Figure 7.

**Figure 8:**
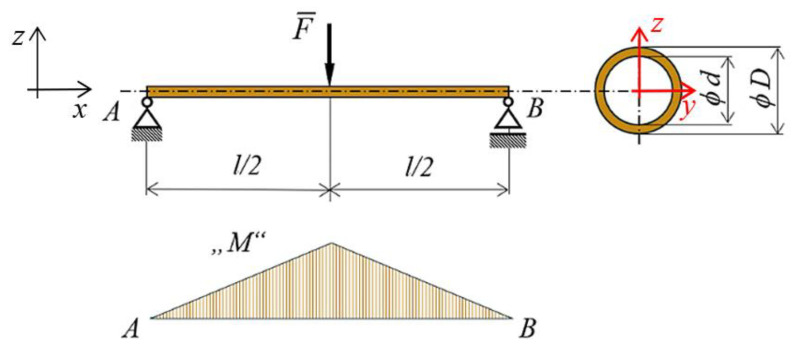
Three-point bending test—principle.

Static calculations of pure bending stresses within linear-elastic range under three-point bending are based on Equations (2) and (3) [37].

(2) $$\sigma =\frac{M}{W}=\frac{M}{I}y\;\;\;(MPa)$$

where *M* is the bending moment, *W* is the section modulus (mm^3^), *I* is the area moment of inertia (also referred to as second moment of area), and *y* is the distance from the neutral axis (mm). For maximal bending, the moment *M* originating under three-point bending tests can be written [38]

(3) $$M=\frac{Fl}{2}\;\;\;(Nm)$$

where *l* is length of the beam between supports and *F* is the applied force, while the maximum values are reached in the middle of the beam, as shown in Figure 8.

If, in addition to the bending moment *M* causing the normal stress, the transverse force *V* also acts in the cross-section, the shear stress also occurs in the cross-section, for which the Jouravski Formula applies [39]:

(4) $$\tau (x)=\frac{V(x)S_{y}^{*}}{bJ_{y}}\;\;\;(MPa)$$

where *V*(*x*) is the transverse force at the point of intersection defined by the coordinate *x*; *b* is the width of the examined cross-section at the point of cut; $J_{y}$ is the axial quadratic moment to the neutral axis; $S_{y}^{*}$ is the static moment of the part of the cross-sectional area at the point where the stress is investigated with respect to the neutral axis [40].

On the other hand, for beams reinforced with lattice and cellular porous structures, there is a critical parameter characterized as the span-to-thickness ratio, often referred to as (*L*/*t*), which plays a role in shear stresses that are neglected in engineering practice if bending loading occurs. It represents the ratio of span length (the distance between supports) to the thickness of a structural element (such as a strut or slab) [41,42,43,44].

In the case of the considered reinforced beams with cellular lattice structures, the highest value is represented by the thickness of the peripheral wall of 2 mm, i.e., the ratio is 170/2 = 85.

**Figure 9:**
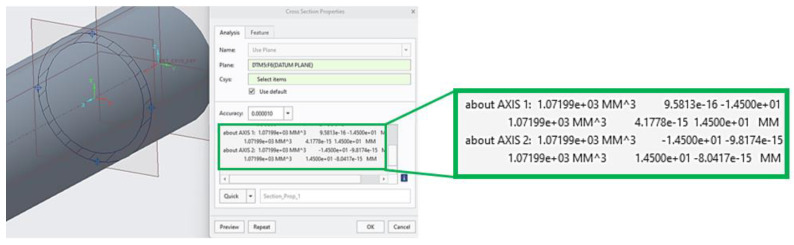
Specification of a structure section modulus within software.

Since the structures are complex and spatial arrangements, it has been advantageous to use software for the specification of a section modulus of the given cross-section area. The verification of the value of the section moduli was carried out on a tube-type sample (see Figure 2 and Figure 8) with diameters *d* = 25 mm and *D* = 29 mm by two approaches. The analytical method is given by Equation (5) and the second method was implemented with computer support using PTC Creo Parametric 8 software, the results of which are shown in Figure 9, while it is clear that the values of both solutions completely match.

(5) $$W_{b}=\frac{\pi (D^{4}-d^{4})}{32\;*\;D}=\frac{\pi (29^{4}-25^{4})}{32\;*\;29}=1071.99\;\;\;({mm}^{3})$$

The obtained section moduli *W_b_* for individual types of samples are presented in Table 3, while B_100 is a bar-type sample (100% filled with material) and T_25 is a tube-type sample.

## 3. Results and Discussion

### 3.1. Bending Stress Evaluation

**Figure 10:**
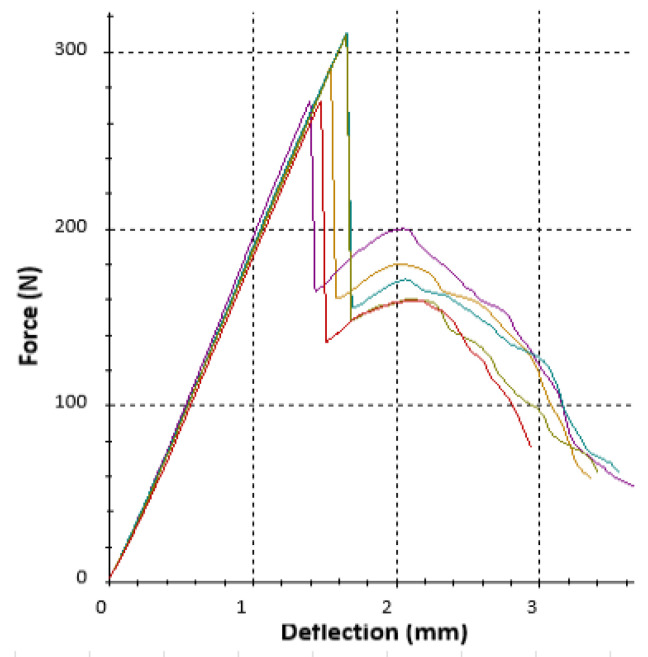
Experimentally obtained force–deflection curves for R_57 specimen (5 repetitions).

Each type of sample (given by structure and volume fraction) was produced in a set of six pieces (one of them was used for preliminary tests), and at the same time, samples of tubular and bar profiles were prepared for testing. During the experiments, the dependence of the force on the deflection was plotted, while an example of the recorded data (for five samples of the same R_57 structure) is shown in Figure 10.

**Figure 11:**
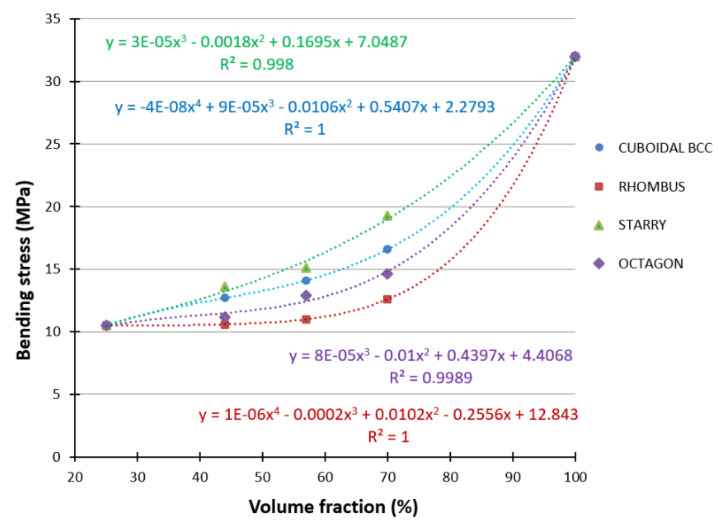
Dependence of bending stress on volume fraction for individual types of structures.

All significant data were listed and subjected to the Grubbs test, which is used to detect outliers in datasets that follow an approximately normal distribution [45]. Outliers were excluded from further processing and then the average value of the maximum force was calculated for the given topology of the structure. This value was the basis for the calculations of maximum bending stresses for individual structures and volume fractions *Vf*. The obtained dependence of the maximum bending stresses on the volume fraction *Vf* is shown in Figure 11, where *Vf* = 25% is for the tube-type specimen and *Vf* = 100% is the stress value corresponding to the rod-type specimen.

The dependence for individual structures can be described by a polynomial function with a high coefficient of determination (R^2^) in the following way:

Starry (R^2^ = 0.998)

*y* = 3 × 10^−5^*x*^3^ − 0.0018 *x*^2^ + 0.1695*x* + 7.0487 (6)

Cuboidal BCC (R^2^ = 1)

*y* = −4 × 10^−8^*x*^4^ + 9 × 10^−5^*x*^3^ − 0.0106*x*^2^ + 0.5407*x* + 2.2793 (7)

Octagon (R^2^ = 0.9989)

*y* = 8 × 10^−5^*x*^3^ − 0.01*x*^2^ + 0.4397*x* + 4.4068 (8)

Rhombus (R^2^ = 1)

*y* = 1 × 10^−6^*x*^4^ − 0.0002*x*^3^ + 0.0102*x*^2^ − 0.2556*x* + 12.843 (9)

When comparing the structures with the same volume ratio *Vf* and the same strut diameter, it is clear from the dependence of the bending stress on the volume fraction *Vf* plotted in Figure 9 that under the given conditions (applied to the selected type of samples made of ABS material using FFF technology), the Starry structure shows the best bending properties and the Rhombus structure shows the worst bending stress properties.

When the structures are arranged according to the bending properties in descending order (best to worst), Starry → Cuboidal BCC → Octagon → Rhombus, it can be seen that structures with a radial distribution alternate in the position with orthogonally distributed structures. It means that the hypothesis about the influence of the radial or orthogonal distribution of the basic cell on the behavior of the lattice structures in bending was not confirmed.

Based on the dependencies in Figure 11, it can also be indicated that the technology plays a significant role in the obtained results, because when comparing the results for tubular samples (*Vf* = 25%) with samples that represent a tube filled with Octagon or Rhombus structures at a volume fraction of *Vf* = 44%, the bending stress reached almost the same values (10.5, 10.6, and 11.2 MPa). It shows that the reinforcing porous structure of the filler did not affect the results achieved and did not strengthen the tube, which should not occur from a mechanical point of view. This statement follows the findings of several research results [46,47,48,49], which have already clarified the role of hollow beam reinforcement and demonstrated that the internal structures play a significant role in increasing the strength through the effective use of the strength of the longitudinal fibers and the restraining effect of the fibers oriented at the hoop circumference. Studies have shown that girders with internal structures offer an effective solution for achieving a maximum load-bearing capacity of the structure while minimizing weight. Their unique design makes them a valuable choice in modern construction where the primary mode of deflection is bending. This research showed that a hollow reinforced beam (HRB) has a higher bending strength than a hollow beam, and it can even achieve a bending strength similar to solid beams, but with a significantly lower weight.

On the other hand, the authors themselves have already proven that the adhesion forces between individual layers of additively produced samples by the FFF method are very small, and therefore, the technology (along with the marginal conditions of production) plays an important role in the resulting mechanical properties of the manufactured components [50].

### 3.2. Energy Absorption and Ductility Assesment

To look closely at a behavior of the selected lattice structures, the ductility and energy absorption during testing were also assessed.

**Figure 12:**
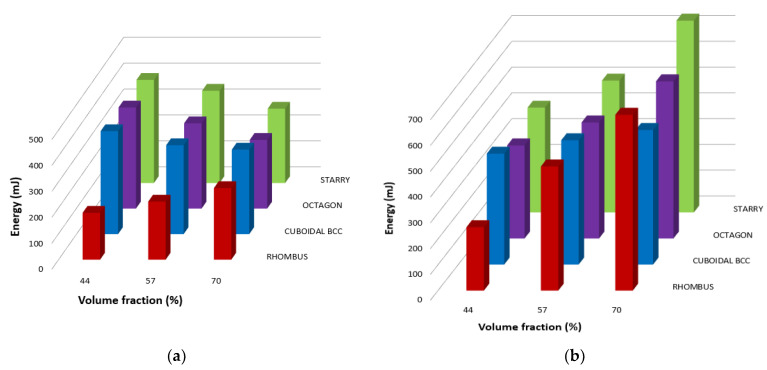
Energy absorption during bending: (**a**) elastic; (**b**) total.

Energy absorption was calculated as the area under the force–deflection curves [51]. The results can be seen in the histograms in Figure 12, where the values of absorbed energy for individual samples within elastic behavior are plotted in Figure 12a and the total amounts of energy absorbed during bending are shown in Figure 12b.

It can be seen from Figure 12 that there is a certain trend in observing the amounts of absorbed energies. It is true that as the volume fraction *Vf* increases, the amount of elastic energy decreases, except for the Rhombus structure, in which it is the opposite (Figure 12a).

An increase in the amount of absorbed energy could be expected with an increase in the volume fraction *Vf* of the material due to higher strengthening of the core of the samples, thus increasing resistance to damage or against deformation until failure, when samples are stressed under bending. The specific behavior of the Rhombus structure in terms of energy absorption with increasing material volume is unexpected even for the authors, and it is not possible to determine unequivocally what part of this behavior is played by the production technology (i.e., adhesion forces between layers), and what part is played by the structure type. However, since all samples were produced under the same conditions, it can be assumed that the topology of the structure (combination of geometry, volume fraction *Vf*, and cell distribution method) is largely involved in this result. Therefore, it can be supposed that in this case, the geometry of other types of samples are closer to a behavior characterized by sharp notches, which can be stress concentrators and cause earlier cracking when the volume fraction *Vf* of the material increases, at which the length of the struts and the angle between them also play a large role. However, this hypothesis will need to be investigated in more detail, which the authors would like to do in the near future [52,53,54].

When looking at the total amount of absorbed energy during bending (Figure 12b), it can be seen that for individual structures, the amount of energy increases with the increase in the volume fraction *Vf*, which is also in accordance with the assumptions and other results of the authors. In all cases, the Starry structures spent the largest amount of energy to reach failure and can be considered the stiffest one among those studied.

Finally, ductility of the beams was assessed via two indices, *µ_d_* and *µ_E_*. The index *µ_d_* represents the ratio between the deflection at the ultimate load *u_u_* (mm) and the deflection at the elastic limit *u_e_* (mm) according to Formula (10) [55]:

(10) $$\mu_{d}=\frac{u_{u}\;}{u_{e}}$$

The ductility index *µ_E_* represents the quotient of the total and elastic energy and it is expressed by Equation (11): [56]

(11) $$\mu_{E}=\frac{1}{2}\left(\frac{W_{tot}}{W_{e}}+1\right)$$

- *W_tot_* is the total energy absorbed by the sample during bending (J);
- *W_e_* is the elastic energy (fraction of total) absorbed by the sample up to the elastic limit (J).

**Figure 13:**
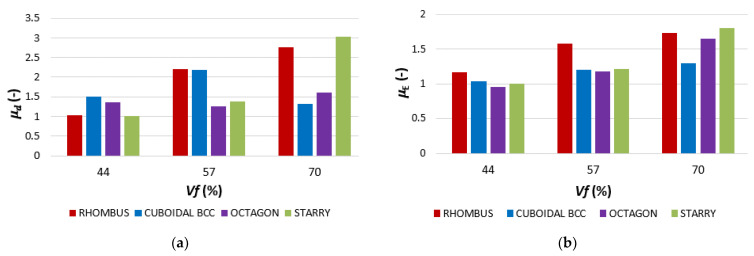
Ductility indices: (**a**) *µ_d_*—based on the deflection value at the proportionality limit; (**b**) *µ_E_*—expressed as the quotient of the total and elastic energy.

The data for calculation of both indexes *µ_d_* and *µ_E_* (Figure 13) were taken from the experimental results (obtained force–deformation dependence), based on which the authors also evaluated the amount of total and elastic energy spent during the sample loading as the corresponding area under the plotted curves.

**Figure 14:**
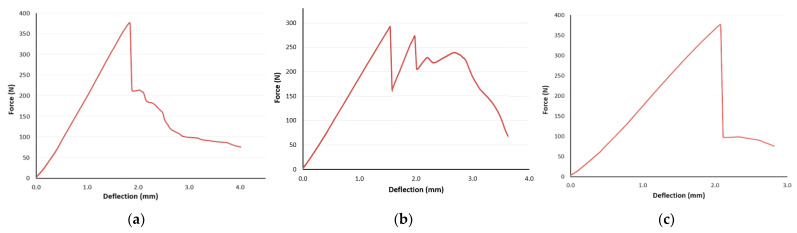
Failure propagation: (**a**) S_44—smooth and slow propagation; (**b)** O_44—stepwise failure; (**c**) C_44—unstable propagation.

The porous samples made from ABS material used within the research showed mostly brittle behavior. Some of them, including the R_57 in Figure 10, showed a sudden drop in applied force after elastic deformation, which can be connected with the theory of failure propagation [57]. Since the cellular structures were studied within the research, it can be said that the type of failure propagation (fast or slow, along the cell boundaries or through the pores, smooth or stepwise, etc.) also affects energy dissipation. Roche et al. [58] proposed five categories for propagation behavior: (1) without failure, (2) unstable propagation, (3) smooth and rapid failure propagation, (4) smooth and slow failure propagation, and (5) stepwise failure propagation. The analyzed samples showed a different failure propagation depending on their topology (the combination of volume fraction *Vf*, cell geometry, and their distribution). The representative responses of samples with volume fraction *Vf* = 44% are shown in Figure 14: (a) S_44—smooth and slow propagation, (b) O_44—stepwise failure, (c) C_44—unstable propagation.

When evaluating the structures’ flexural behavior, it was found that both the factors of volume fraction *Vf* and the type of structure contribute to the results [59], and the combination of these factors makes it impossible to observe any unequivocal regularities in behavior trends in the assessment of ductility. Based on the obtained ductility indices, it can be concluded that the Rhombus structure appears to be the best in terms of ductility; however, the structure showed the lowest values of bending stresses. Therefore, from a synergic point of view of both factors, stress and ductility, the Starry structure appears to be the most suitable for application in rotating components stressed by bending.

## 4. Conclusions

Lattice porous materials with regularly distributed cells have become a new challenge not only for many scientific studies but also for many industrial and household applications because of their outstanding properties. The production of such sophisticated components has been enabled thanks to additive technologies. However, a wide variability of materials, types of porous structures, as well as 3D printers and manufacturing conditions force researchers to investigate properties of materials under various loading in order to use the most appropriate combination under the given conditions.

The presented study has aimed to compare the bending behavior of cylindrically shaped lattice specimens with radially and orthogonally arranged cells made of ABS material. Four types of structures were evaluated: Rhombus, Cuboidal BCC, Octagon, and Stary in the three volume fractions *Vf* = 44, 57, and 70%, together with tubular and bar-shaped samples.

- The results showed that the Starry structure reached the highest values of stress in all volume fractions *Vf* and it can absorb the highest amount of energy.
- When the structure types were arranged according to their bending properties in descending order (best to worst), it could be seen that structures with a radial distribution alternate in position with orthogonally distributed structures. It means that the hypothesis about the influence of the radial or orthogonal distribution of the basic cell on the behavior of the lattice structures in bending was not confirmed.
- The samples with lattice structures made from ABS material by FFF technology used for the research showed mostly a brittle fracture behavior, while the failure propagation differs and it is affected by their topology (a combination of geometry, material volume fraction *Vf*, and cell distribution).
- In addition, the results showed that technology plays a significant role, since no difference (or very little) was visible in the obtained values when comparing the bending properties of the tube-shaped sample without structure and with structure (at *Vf* = 44%), and no difference (or really very small) in the obtained values was visible.
- Ductility indices *µ_d_* (based on the deflection value at the proportionality limit) and *µ_E_* (expressed as the quotient of the total and elastic energy) were also evaluated. Based on the obtained ductility indices, it can be concluded that the Rhombus structure appears to be the best; however, the structure showed the lowest values of bending stresses. Therefore, from a synergic point of view of both factors, stress and ductility, the Starry structure appears to be the most suitable for application in rotating components stressed by bending.

## Figures and Tables

**Table 1:** The basic characteristics of individual basic cells.

Structure Type	Volume Fraction *Vf* (%)	Sample Designation	Cell Sizes * (mm)	Strut Diameter (mm)	Number of Pieces
Tube type	25	T_25	*-*	-	6
Cuboidal BCC	44	C_44	*x*, *y*, *z* = 5 mm	1	6
Rhombus		R_44	*x =* 7 mm; *y =* 7 mm; *z* = 5.5 mm; *φ =* 45°		6
Starry		S_44	*x =* 5 mm; *y =* 9 mm; *z =* 7.5		6
Octagon		O_44	*x =* 5 mm; *y =* 7 mm; *z =* 6 mm		6
Cuboidal BCC	57	C_57	*x*, *y*, *z* = 5 mm	1.4	6
Rhombus		R_57	*x* = 7 mm; *y* = 7 mm; *z* = 5.5 mm; *φ* = 45°		6
Starry		S_57	*x* = 5 mm; *y* = 9 mm; *z* = 7.5		6
Octagon		O_57	*x* = 5 mm; *y* = 7 mm; *z* = 6 mm		6
Cuboidal BCC	70	C_70	*x, y, z* = 5 mm	1.8	6
Rhombus		R_70	*x =* 7 mm; *y =* 7 mm; *z =* 5.5 mm; *φ* = 45°		6
Starry		S_70	*x* = 5 mm; *y* = 9 mm; *z* = 8		6
Octagon		O_70	*x* = 5 mm; *y* = 7 mm; *z* = 5.5 mm		6
Bar-type	100	B_100	*-*	-	6

* x—Rotation Axis.

**Table 2:** The basic characteristics of the White EasyABS [27].

Characteristics	Designation	Unit	Value
Ultimate tensile strength	Rm	MPa	43
Young’s modulus	*E*	MPa	2140
Poisson number	*ν*		0.394
Density	*ρ*	g/cm^3^	1.05

**Table 3:** Section moduli used for bending stress calculation.

Sample	Section Moduli (mm^3^)
C_44	1212.29
C_57	1352.12
C_70	1542.77
R_44	1463.70
R_57	1691.13
R_70	1937.27
S_44	1260.66
S_57	1578.53
S_70	1773.23
O_44	1390.69
O_57	1608.98
O_70	1968.59
B_100	2394.38
T_25	1071.99

## Data Availability

Data are contained within the article.
